# Population-Level Impact of Ontario’s Infant Rotavirus Immunization Program: Evidence of Direct and Indirect Effects

**DOI:** 10.1371/journal.pone.0154340

**Published:** 2016-05-11

**Authors:** Sarah E. Wilson, Laura C. Rosella, Jun Wang, Nicole Le Saux, Natasha S. Crowcroft, Tara Harris, Shelly Bolotin, Shelley L. Deeks

**Affiliations:** 1 Public Health Ontario, Toronto, Ontario, Canada; 2 Dalla Lana School of Public Health, University of Toronto, Toronto, Ontario, Canada; 3 Institute for Clinical Evaluative Sciences, Toronto, Ontario, Canada; 4 Division of Infectious Disease, Children’s Hospital of Eastern Ontario, Ottawa, Ontario, Canada; 5 Department of Pediatrics, University of Ottawa, Ottawa, Ontario, Canada; 6 Department of Laboratory Medicine and Pathobiology, University of Toronto, Toronto, Ontario, Canada; New York City Department of Health and Mental Hygiene, UNITED STATES

## Abstract

**Objective:**

To evaluate the direct and indirect population impact of rotavirus (RV) immunization on hospitalizations and emergency department (ED) visits for acute gastroenteritis (AGE) in Ontario before and after the publicly-funded RV immunization program.

**Methods:**

Administrative data was used to identify ED visits and hospitalizations for all Ontarians using ICD-10 codes. We used two outcome definitions: RV-specific AGE (RV-AGE) and codes representing RV-, other viral and cause unspecified AGE (“overall AGE”). The pre-program and public program periods were August 1, 2005 to July 31, 2011; and August 1, 2011 to March 31, 2013, respectively. A negative binominal regression model that included the effect of time was used to calculate rates and rate ratios (RRs) and 95% confidence intervals (CIs) for RV-AGE and overall AGE between periods, after adjusting for age, seasonality and secular trends. Analyses were conducted for all ages combined and age stratified.

**Results:**

Relative to the pre-program period, the adjusted RRs for RV-AGE and overall AGE hospitalizations in the public program period were 0.29 (95%CI: 0.22–0.39) and 0.68 (95%CI: 0.62–0.75), respectively. Significant reductions in RV-AGE hospitalizations were noted overall and for the following age bands: < 12 months, 12–23 months, 24–35 months, 3–4 years, and 5–19 years. Significant declines in overall AGE hospitalizations were observed across all age bands, including older adults > = 65 years (RR 0.80, 95%CI: 0.72–0.90). The program was associated with adjusted RRs of 0.32 (95% CI: 0.20–0.52) for RV-AGE ED visits and 0.90 (95% CI: 0.85–0.96) for overall AGE ED visits.

**Conclusions:**

This large, population-based study provides evidence of the impact of RV vaccine in preventing hospitalizations and ED visits for RV-AGE and overall AGE, including herd effects.

## Introduction

Prior to vaccine introduction, infection with rotavirus (RV) was a common cause of acute gastroenteritis (AGE) in Canadian children, responsible for 10–40% of all childhood gastroenteritis and associated with more health care resource utilization than other causes of AGE [1]. During that time, it was estimated that among Canadian children with RV, one-third would see a physician, 15% would visit an emergency department (ED) and 7% required hospitalization [2]. Approximately two-thirds of hospitalizations occurred among children under 2 years of age [2,3]. In addition, the societal (indirect) costs are substantial [4–6] and this has been a key consideration in RV immunization program decision-making in Canada [7].

In Canada, two vaccines are authorized for use: RotaTeq^®^ (RV5, Merck Canada Inc.) since August 2006 [8] and Rotarix^(™)^ (RV1, GlaxoSmithKline Inc.) as of October 2007 [9]. The Canadian National Advisory Committee on Immunization first recommended RV vaccine in January 2008 [10], with expanded guidance in July 2010 [11]. On August 8, 2011, Ontario became one of the first Canadian provinces to implement a universal, publicly-funded RV immunization program, using Rotarix^®^ vaccine at 2 and 4 months of age. Immunization coverage for the first year of Ontario’s program has been estimated at approximately 80% based on vaccine distribution data [12]. As of March 2016, ten of Canada’s 13 provinces and territories (P/Ts) have introduced publicly-funded RV vaccine programs [13].

Other international jurisdictions with RV immunization programs have demonstrated a rapid and dramatic impact on healthcare utilization, observing reductions in hospitalizations by as much as 94% [14–24] with program impact demonstrated as early as one to two years following implementation. A reduction in all cause AGE and RV-AGE has also been seen in non-immunized cohorts, suggesting a herd effect of RV vaccine [25–28].

Our objective for this study was to evaluate the population level direct and indirect effects of RV immunization in Ontario on health services utilization, hospitalizations and ED visits, for AGE since the introduction of the publicly-funded RV immunization program.

## Methods

### 1) Program impact

#### (i) Study population and setting

We conducted a retrospective longitudinal population-based cohort study examining healthcare utilization for AGE between the period of August 1, 2005 and March 31, 2013 among all Ontarians with a valid health card for the Ontario Health Insurance Plan (OHIP). OHIP covers almost all of Ontario’s approximately 13.5 million residents, except for newcomers who have resided in the province for less than three months and refugees covered under federal health programs. There is no parallel private delivery of health services in Ontario for hospitalizations or ED visits. These datasets were linked using unique encoded identifiers and analyzed at the Institute for Clinical Evaluative Sciences (ICES). This study was approved by the institutional review boards at Sunnybrook Health Sciences Centre and Public Health Ontario in Toronto, Canada. Analyses were conducted using SAS Enterprise Guide 6.1 (SAS Institute, Cary, NC).

#### (ii) Data sources

Individual-level hospitalizations and ED visits were identified using the Discharge Abstract Database (DAD) of the Canadian Institutes for Health Information (CIHI) and the National Ambulatory Care Reporting System (NACRS), respectively using International Classification of Diseases, Tenth Edition (ICD-10) diagnostic codes. We counted all events (hospitalizations and ED visits), to examine program impact on the different aspects of the healthcare system. Validation of the ICD code for RV-AGE has been completed in the United States (US) and Australia and has been shown to have a high positive predictive value, but low sensitivity since diagnostic testing for AGE illness is not always performed [29–31]. As such, similar to others [26,27], we assessed two distinct outcomes: (1) events with the diagnostic code specific to RV-AGE (*rotaviral enteritis*, A08.0) and (2) events with either the RV-AGE code or a non-specific code for AGE (hereafter referred to as “overall AGE”). To address this, studies evaluating RV vaccine program impact have included additional AGE diagnostic codes. Our study used the following ICD-10 codes: *rotaviral enteritis* (A08.0), *other viral gastroenteritis* (A08.3), *viral intestinal infection*, *unspecified* (A08. 4), *other specified intestinal infections* (A08.5), *other gastroenteritis and colitis of infectious and unspecified origin* (A09) and *noninfective gastroenteritis and colitis*, *unspecified* (K52.9). The final code (K52.9) was added after obtaining documentation confirming a change in directive for the classification of unspecified gastroenteritis within ICD-10, which is described in detail elsewhere [32,33]. Only the diagnostic code listed as the most responsible for the patient’s hospitalization or ED visit (diagnosis type M category) was used for outcome ascertainment. Annual population estimates and ages were obtained from the Registered Persons Database (RPDB).

#### (iii) Statistical analysis

The study was divided into two time periods: pre-program (August 1 2005-July 31 2011) and the period following the introduction of the publicly-funded program (hereafter referred to as “public program period”) (August 1 2011-March 31, 2013). Although RV vaccines were available for private purchase starting in August 2006, private market sales data was obtained from the respective manufacturers for the period preceding introduction of the public program and when both vaccines were available for purchase (January 1, 2008 to July 31, 2011) and coverage was estimated to be low (< 15%). Other jurisdictions, including those in Canada [34], have found limited [35] or no [34] reduction in RV-AGE hospitalizations during periods of low to moderate RV vaccine uptake; thus, we included the years when RV vaccine could be purchased through out of pocket payment (as opposed to publicly funded) within the pre-program period. However, we included a broad time horizon within our pre-program period to mitigate any dilution of effect that could result from including private vaccine availability within the reference period.

Crude age-specific average monthly rates of AGE using the two outcome definitions (RV-AGE and RV and unspecified AGE) were calculated separately for hospitalizations and ED visits for the two periods using the following age strata: < 12 months, 12–23 months, 24–35 months, 3–4 years, 5–19 years, 20–44 years, 45–64 years, ≥ 65 years. Due to the prominent seasonality of RV infections and uneven observation time across the periods, average monthly rates were calculated by dividing the number of observed events by the number of months within each time period.

We used a negative binominal regression model that included the effect of time to assess the trend in monthly rates of AGE adjusting for age, secular trends and seasonality. Secular trends were adjusted for with the use of a linear term. Seasonality was adjusted by using groupings of 3 months with the fall (September, October, November) set as the reference period based on the historical trends of lowest RV healthcare utilization in Ontario occurring in these months. Alternative adjustment of seasonality by individual month was also explored in a sensitivity analysis. A variable indicating vaccine period was used to determine the impact of the public program, in comparison to the reference period (pre-program) on AGE rates. All descriptive and regression analyses were conducted for all ages and also age stratified. Additionally, we tested for differences in median age for AGE before and after the program’s introduction using the Kruskal-Wallis test.

### 2) RV immunization coverage

In order to assist in the interpretation of our findings, RV vaccine coverage during the public program was estimated. For the period of August 2011 to March 2013, the net number of doses distributed for publicly-funded RV1 vaccine was obtained from the Ontario Government Pharmacy and Medical Supply Service (OGPMSS). Net vaccine distribution data are adjusted for wasted or reusable vaccine returned to OGPMSS. Only annual coverage for 2012 was estimated given the instability of distribution data during early program implementation (August to December 2011). To estimate full series coverage we divided the total number of RV1 doses by two. Ontario population estimates for infants under 12 months from Statistics Canada comprised the denominator data.

## Results

During our approximate eight year study period there were 2,465 hospitalizations and 373 ED visits for RV-AGE, and 127,294 hospitalizations and 734,130 ED visits for overall AGE.

In the pre-program period, the highest age-specific rates of RV-AGE and overall AGE occurred among children 12–23 months of age, for both hospitalizations and ED visits (Table 1). Since the implementation of the publicly-funded program, depending on outcome definition and location of care, children < 12 months (overall AGE hospitalizations and RV-AGE ED visits) or 12–23 months (RV-AGE hospitalizations and overall AGE ED visits) had the highest age-specific (unadjusted) average monthly rate. The unadjusted average age-specific monthly rate of RV-AGE, as well as overall AGE hospitalizations and ED visits decreased in all age cohorts with events, with the exception of 20–44 year old adults for overall AGE ED visits (Table 1). The reduction in the average age-specific monthly rate of RV-AGE hospitalizations occurred most dramatically among infants less than 12 months of age with a 6 fold reduction between the private purchase (0.52 per 10,000 population) and public program (0.08 per 10,000 population) periods.

**Table 1:** Unadjusted average monthly rate (per 10,000 population) of RV-AGE and overall AGE hospitalizations and ED visits before and after RV immunization program implementation, August 1, 2005 to March 31, 2013: Ontario, Canada.

	Hospitalizations				ED visits			
	RV-AGE		Overall AGE		RV-AGE		Overall AGE	
Age group	Pre-Program	Public Program	Pre-Program	Public Program	Pre-Program	Public Program	Pre-Program	Public Program
<1 year	0.52	0.08	4.55	2.57	0.08	0.04	41.83	31.20
12–23 months	0.76	0.17	5.90	2.43	0.09	0.02	52.02	35.90
24–35 months	0.37	0.14	3.60	1.59	0.06	0.02	30.02	21.78
3–4 years	0.18	0.06	1.95	0.92	0.01	0.00	16.80	13.17
5–19 years	0.01	0.00	0.46	0.30	0.00	0.00	5.43	5.16
20–44 years	0.00	0.00	0.73	0.41	0.00	0.00	5.04	5.13
45–64 years	0.00	0.00	1.10	0.61	0.00	0.00	3.25	3.21
> = 65 years	0.00	0.00	2.45	1.69	0.00	0.00	6.52	6.03

Among RV-AGE hospitalizations, median age significantly increased from 1.8 years pre-program to 2.3 years in the public period (p = 0.008). The median age among RV-AGE ED visits increased following the introduction of the program, from 1.7 to 1.9 years, but this was not significant (p = 0.39). Across both periods, outcome definitions and location of care, males accounted for approximately half (40 to 56%) of all events, with the exception of RV AGE ED visits during the public period where it was 75%. However, there were only 32 events during this period increasing the likelihood of chance variation in the proportion that was male.

**Fig 1:**
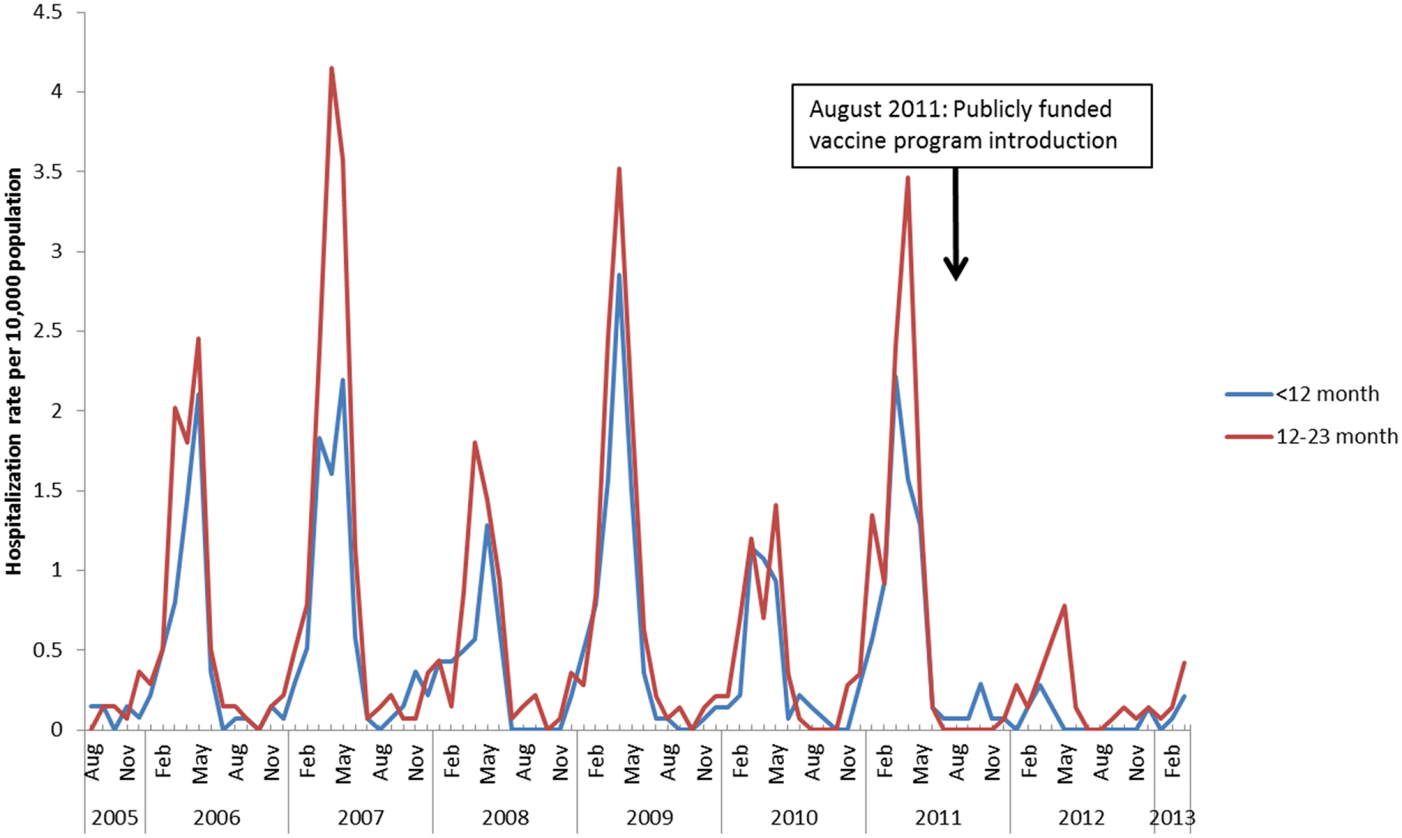
Hospitalization rates for RV-AGE among children < 24 months of age per 10,000 population, by month and year, August 1, 2005-March 31, 2013: Ontario, Canada.

**Fig 2:**
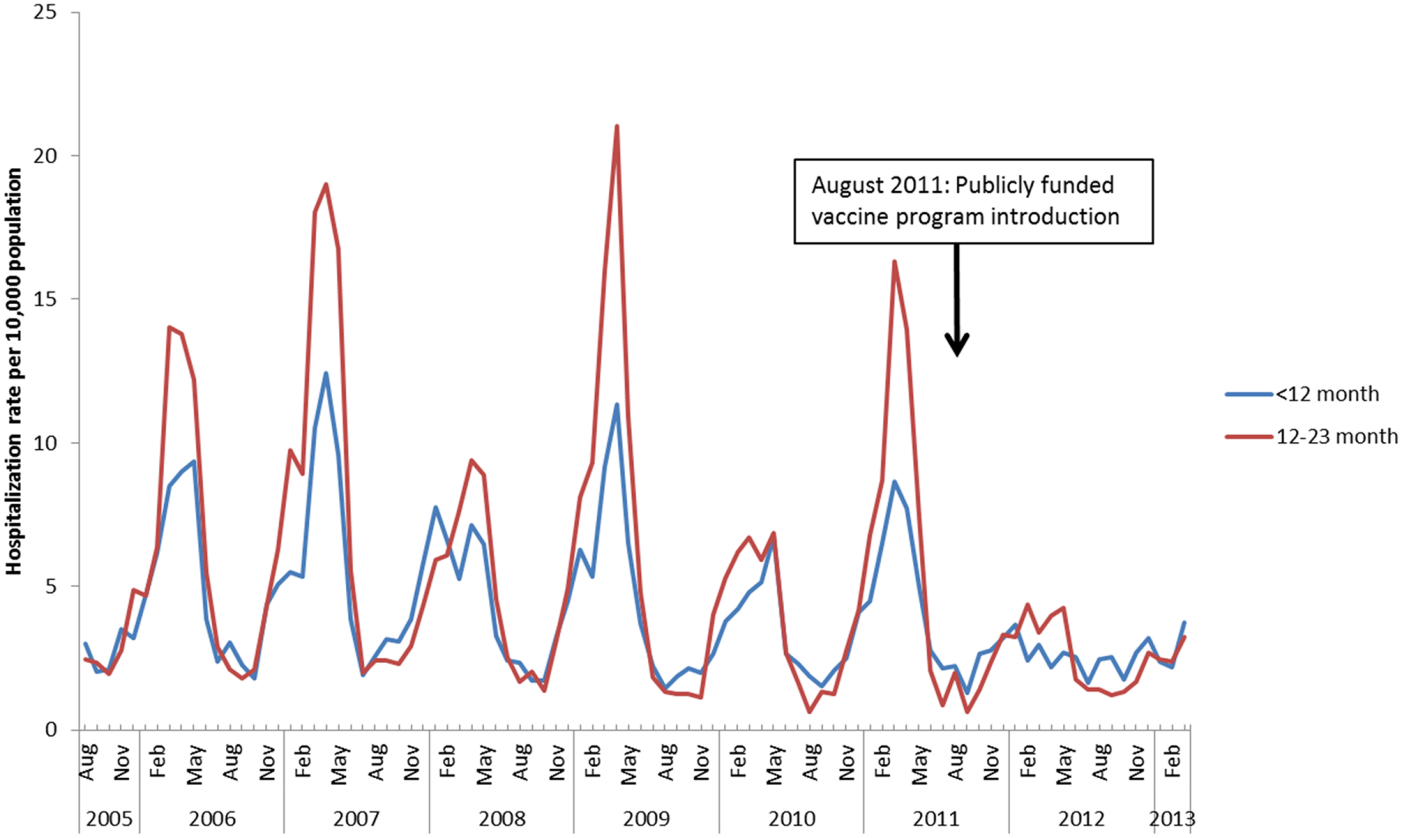
Hospitalization rates for overall AGE among children < 24 months of age per 10,000 population, by month and year, August 1, 2005-March 31, 2013: Ontario, Canada.

The prominent seasonality of RV-AGE and overall AGE hospitalizations and the unadjusted reduction in these events among children under 2 years of age following program implementation is shown in Figs 1 and 2. These figures also demonstrate the periodicity of RV infections, with a pattern of high followed by low burden seasons.

### RV vaccine program impact on AGE hospitalizations

Ontario’s RV program was associated with significantly reduced RV-AGE and overall AGE hospitalizations, in both the unadjusted analyses and after adjustment for age, seasonality and secular trends (Table 2). Age-stratified analyses suggest both direct and indirect (herd) vaccine effects.

**Table 2:** Rate ratios (unadjusted and adjusted) for changes in RV AGE and overall AGE hospitalizations associated with publicly funded RV immunization program: Ontario, Canada.

	RV AGE	Overall AGE
	RR (95% CI)	RR (95% CI)
Unadjusted	0.23 (0.14–0.39)	0.50 (0.43–0.59)
Adjusted*	0.29 (0.22–0.39)	0.68 (0.62–0.75)
Age stratified**		
<1 year	0.21 (0.11–0.40)	0.80 (0.65–0.99)
12–23 months	0.27 (0.16–0.48)	0.70 (0.53–0.92)
24–35 months	0.48 (0.27–0.87)	0.70 (0.52–0.93)
3–4 years	0.31 (0.16–0.60)	0.65 (0.5–0.84)
5–19 years	0.25 (0.13–0.50)	0.70 (0.61–0.80)
20–44 years	0 (0-.)	0.62 (0.51–0.76)
45–64 years	0 (0-.)	0.62 (0.51–0.74)
> = 65 years	0.57 (0.10–3.15)	0.80 (0.72–0.90)

*Adjusted for age, seasonality and secular trends

**Adjusted for seasonality and secular trends.

During the public program period, the greatest reduction in the adjusted rate of RV-AGE hospitalizations occurred in infants <1 year (RR 0.21, 95% CI 0.11–0.40). Children 12–23 months of age had a reduction of 73% (RR 0.27, 95% CI 0.16–0.48). Statistically significant reductions in RV-AGE hospitalizations were also noted for children 24–35 months, 3–4 years of age, and 5–19 years of age. Hospitalizations due to RV-AGE declined to a larger extent (71%) than overall AGE hospitalizations (32%). When examining age-specific effects for overall AGE hospitalizations, significant reductions of between 20–38% were found across all age groups, including seniors 65 years of age and older (RR 0.80, 95% CI 0.72–0.90).

### RV vaccine program impact on AGE ED visits

The public program period was associated with significant decreases of 68% for RV-AGE ED visits (RR 0.32, 95% CI 0.20–0.52) and 10% for overall AGE ED visits (RR 0.90, 95% CI 0.85–0.96) in the adjusted analysis for the Ontario population, with both direct and indirect effects observed (Table 3). Age-specific RV-AGE ED visits were significantly reduced by 77% among toddlers 12–23 months old (RR 0.23, 95% CI 0.08–0.63), children 3–4 years of age (RR 0.12, 95% CI 0.02–0.60) and 5–19 years of age (RR 0.16, 95% CI 0.04–0.60). Overall AGE ED visits were significantly reduced by 18 and 19% among young children 24–35 months and 3–4 years of age, respectively.

**Table 3:** Rate ratios (unadjusted and adjusted) for changes in RV AGE and overall AGE ED visit associated with publicly funded RV immunization program: Ontario, Canada.

	RV AGE	Overall AGE
	RR (95% CI)	RR (95% CI)
Unadjusted	0.27 (0.13–0.56)	0.76 (0.64–0.89)
Adjusted*	0.32 (0.20–0.52)	0.90 (0.85–0.96)
Age stratified**		
<1 year	0.76 (0.30–1.91)	0.90 (0.78–1.04)
12–23 months	0.23 (0.08–0.63)	0.84 (0.69–1.01)
24–35 months	0.60 (0.15–2.36)	0.82 (0.68–0.98)
3–4 years	0.12 (0.02–0.60)	0.81 (0.69–0.94)
5–19 years	0.16 (0.04–0.60)^ǂ^	0.91 (0.82–1.01)^ǂ^
20–44 years	0.30 (0.02–4.58)	1.02 (0.93–1.11)
45–64 years	0 (0-.)^ǂ^	1.03 (0.94–1.13)^ǂ^
> = 65 years	0.80 (0.05–14.15)	0.99 (0.90–1.10)

*Adjusted for age, seasonality and secular trends

**Adjusted for seasonality and secular trends

^ǂ^ Due to small cell sizes for these age strata, a warning message from SAS was issued noting it had to increase its standard iterations in order to generate the RR estimate via Maximum Likelihood estimation. In doing so, the convergence criterion was lowered. Caution is advised when interpreting these RR estimates.

#### RV immunization coverage

In 2012, the first complete year of the public program period, RV vaccine coverage (series completion) among infants less than 1 year of age, was estimated to be 87%.

## Discussion

This large, population-based study provides robust estimates of the impact of a publicly-funded RV immunization program in preventing hospitalizations and ED visits for RV-AGE and overall AGE. The impact of the public program translated into a reduction in hospitalizations by up to 79% for RV-AGE hospitalizations in age cohorts ranging from < 12 months to 19 years of age compared to the pre-program era. Age groups ineligible for the program were also found to have a significant reduction in overall AGE, particularly for hospitalizations, suggesting an indirect (herd) effects of the publicly-funded program only 1.5 years (20 months) after implementation.

This study adds to the accumulating global literature demonstrating the impressive impact of RV immunization programs on healthcare utilization for AGE, but is among the first to demonstrate program impact in a Canadian province or territory [23]. The magnitude of AGE reduction we observed in vaccine eligible cohorts during the public program is similar in magnitude to what other investigators have found, with declines of 50 to more than 80% for RV-AGE hospitalizations in children under 5 years of age [14,16,17,19] and declines of 17 to 55% for all cause AGE hospitalizations in the same age group [14,16,36–39] in the first several years following program introduction.

Our focus on population impact on both ED visits and hospitalizations, including all age groups, allowed us to explore health system and indirect effects of Ontario’s RV program. Similar to other investigators, we confirmed herd effects among older children never eligible for RV vaccine [26,27,37,40]. Our lack of individual-level immunization status precluded us from exploring program impact among unimmunized age-eligible children which has been observed elsewhere [28]. Our finding that Ontario’s publicly-funded program was associated with a reduction in AGE hospitalizations for adult age groups, adds to an emerging literature confirming benefits in adults. In the United States, Lopman and coauthors found significant reductions in both RV-AGE and cause-unspecified AGE hospitalizations among those 5–14 and 15–24 years, with a non-significant reduction in older age groups [26]. A later analysis extending the post-vaccine period to include 2008–2010, confirmed the above findings and also found indirect effects for adults 25–44 years of age for cause-unspecified AGE hospitalizations (RR 0.94, 95% CI 0.90–0.98) [27]. Studies examining the percent positive for RV among adult stool specimens in a large hospital setting in Chicago, USA [41] and in Queensland, Australia where laboratory-confirmed rotavirus disease became notifiable in 2005 [25] also support indirect effects among adults.

The population-based nature of this study, ability to separate sector-specific health care utilization, age-specific utilization and its associated large sample size, which facilitated age-stratified analyses, are important strengths. Our approach to outcome ascertainment utilized a validated ICD code for RV-AGE [29–31], in addition to a broader outcome definition encompassing other viral and unspecified etiologies for AGE, but which did not include ICD codes associated with bacterial enteritis, parasitic disease and other confirmed etiologies for AGE. Although several investigators have measured RV vaccine program impact using a larger range of gastroenteritis diagnostic codes, others have used a more selective range of codes similar to our approach [26,27].

There are several limitations which deserve mention. Our primary objective was to determine whether there was early impact of Ontario’s program and as a consequence only one complete RV season was included in our analyses. In addition, our interpretation of early impact may be complicated by the pattern of high and low RV years, outside of seasonal oscillations and possible secular trends which were both controlled for in the regression model. We intend to extend these analyses, adding additional years of data associated with the publicly-funded program to confirm these findings and to determine whether the extent of direct and indirect protection is sustained. Next, as with any study utilizing administrative data, there is the possibility of misclassification. This is particularly true for AGE where clinical management is largely syndromic and laboratory testing infrequently completed [42]. Valid estimates of RV vaccine program impact using administrative data are dependent on the comparability of hospital discharge coding practices and RV stool testing patterns pre- and post- program implementation. Jayasinghe and Macartney [31] examined hospitalization ICD-10 data and laboratory testing in a large tertiary pediatric hospital in Australia pre-and post- vaccine program implementation. They found that the sensitivity and positive predictive value of the RV-specific code (A08.0) had not significantly changed following program implementation despite evidence of greater RV stool testing [31]. Finally, we included the period of private vaccine purchase within our reference period, rather than excluding these years from our analysis. This may have attenuated the magnitude of program impact that we observed.

## Conclusions

This large, population-based cohort study provides evidence of the impact of a publicly funded RV immunization program in preventing hospitalizations and ED visits for AGE at the population level, including herd effects, only 20 months following program implementation. This study adds to the accumulating literature on the impressive impacts of RV vaccine programs on healthcare utilization, particularly in developed countries where this may be an important rationale for program implementation. This study will be of interest to vaccine decision makers in jurisdictions that have yet to implement publicly funded RV programs within their routine immunization schedules.
